# Supplementary material for: Separation of Mercury(II) from Industrial Wastewater through Polymer Inclusion Membranes with Calix[4]pyrrole Derivative
Source: Membranes (Basel). 2022 Apr 30;12(5):492. doi: 10.3390/membranes12050492 (PMC9147343; doi:10.3390/membranes12050492)
Supplement: Supplementary file 1 [file membranes-12-00492-s001.zip › membranes-1676312-supplementary.pdf]

membranes

MDPI

Supplementary material

Separation of mercury(II) from industrial wastewater through polymer inclusion membranes with calix[4]pyrrole derivative

Iwona Zawierucha [1,*], Anna Nowik-Zajac [1], Jakub Lagiewka [1] and Grzegorz Malina [2]

1 Institute of Chemistry, Jan Dlugosz University in Czestochowa; Czestochowa 42-200, Poland; a.zajac@ujd.edu.pl, jakub.lagiewka@doktorant.ujd.edu.pl
2 Department of Hydrogeology and Engineering Geology, AGH University of Science and Technology, Mickiewicza 30, Cracow 30-059, Poland; gmalina@agh.edu.pl
* Correspondence: i.zawierucha@ujd.edu.pl; Tel.: +48 883 842 222

The "Supplementary materials" section contains the NMR and FT-IR spectra of synthesized *meso*-octamethylcalix[4]pyrrole.

^{1}H NMR (400 MHz, $CDCl_3$, δ, ppm) (Figure 1A): 6.97 (s, 1H, NH), 5.88 (d, 2H, PyH, J = 2.7 Hz), 1.49 (s, 6H, CH_3). ^{13}C NMR (100 MHz, $CDCl_3$, δ, ppm) (Figure 1B): 29.10 (CH_3), 35.19 ($C(CH_3)_2$), 102.83 (ArH), 138.43 (Ar).

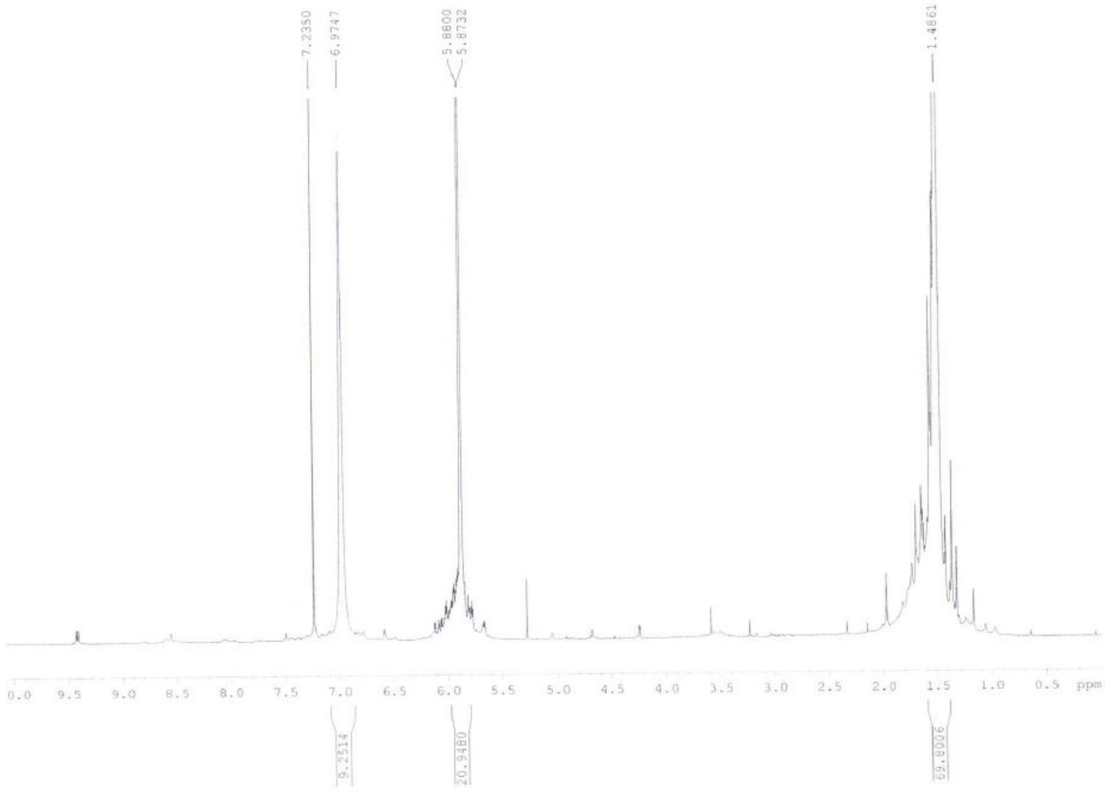

A

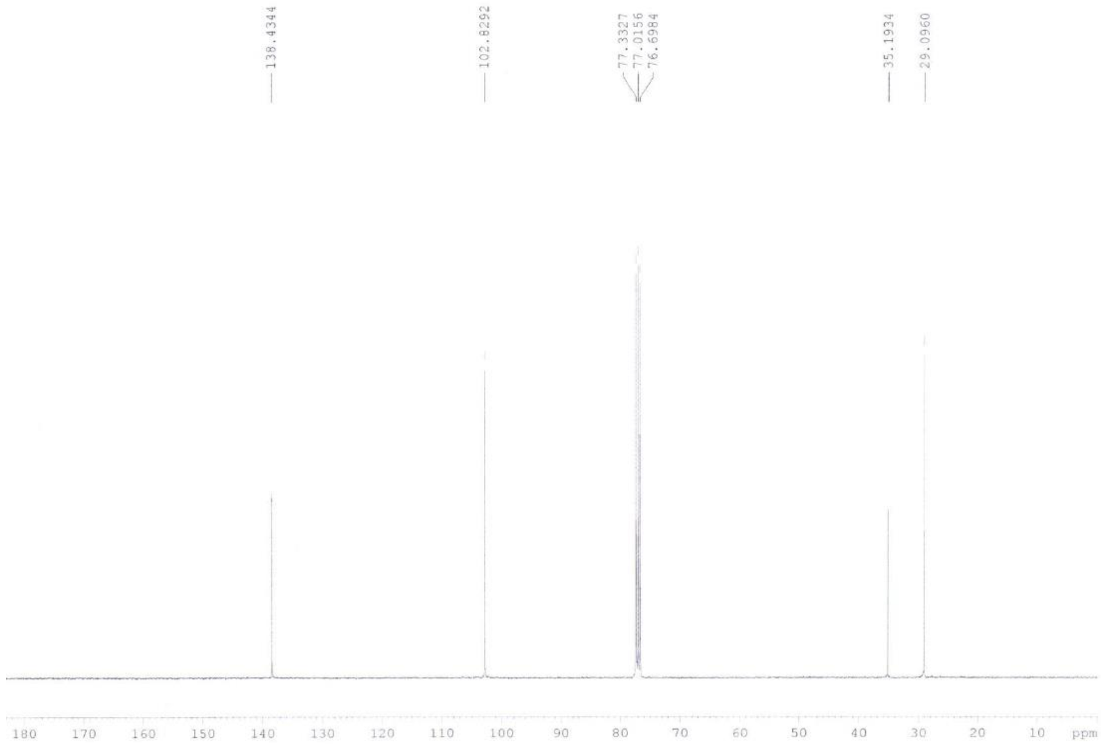

B

Figure S1. (**A**) ^{1}H NMR spectrum of the *meso*-octamethylcalix[4]pyrrole; (**B**) ^{13}C NMR spectrum of the *meso*-octamethylcalix[4]pyrrole.

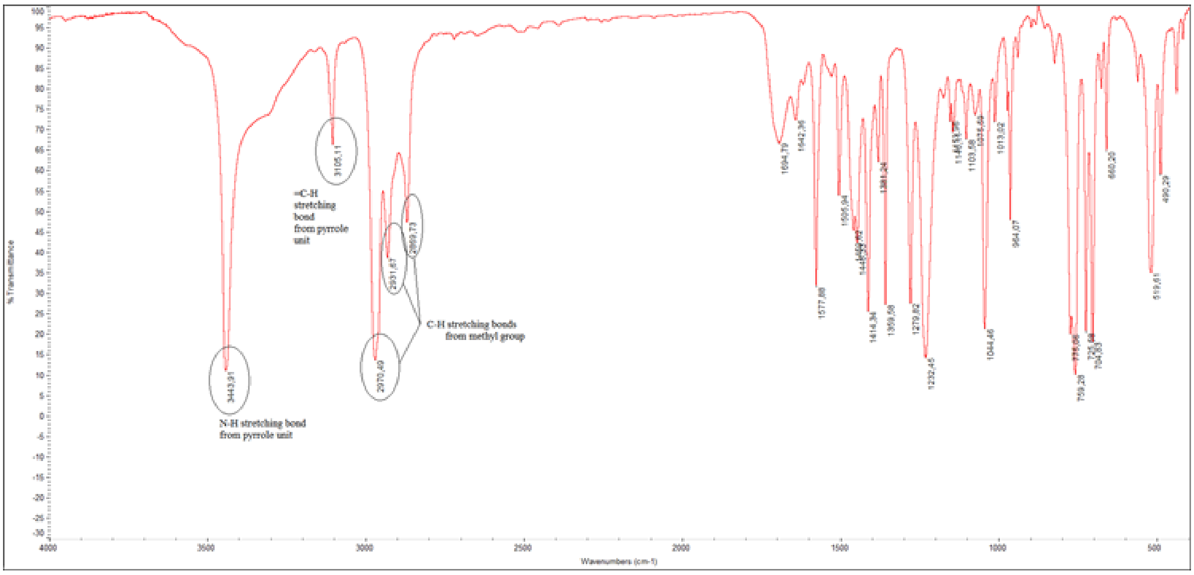

Figure S2. FT-IR spectrum of *meso*-octamethylcalix[4]pyrrole.

For pyrrole units, one strong and sharp peak at 3443 cm^{-1} responds to N-H stretching while a less intensive peak at 3105 cm^{-1} corresponds with =C-H stretching from an aromatic ring. The successive peaks at: 2970, 2934 and 2870 cm^{-1} reflect the C-H stretching

bonds from methyl groups. Stretching bonds from pyrrole units and methyl groups indicate presence of *meso*-octamethylcalix[4]pyrrole.
